# Pragmatic recruitment strategies for a text-based smoking cessation intervention for sexual and gender minority groups

**DOI:** 10.1186/s12982-025-01117-0

**Published:** 2025-11-24

**Authors:** Samuel Tundealao, Marie Compaore Oubda, Rebecca Klaff, Belinda Reininger, Lorna McNeill, Irene Tamí-Maury

**Affiliations:** 1 Department of Epidemiology and Biostatistics, Rutgers School of Public Health, Piscataway, NJ USA; 2 Department of Epidemiology, School of Public Health, The University of Texas Health Science Center at Houston, Houston, TX USA; 3 Department of Health Promotion and Behavioral Sciences, School of Public Health, The University of Texas Health Science Center at Houston, Houston, TX USA; 4 Department of Health Disparities, The University of Texas MD Anderson Cancer Center, Houston, TX USA

**Keywords:** SGM, Smoking cessation, Recruitment strategies, Enrollment

## Abstract

**Background:**

SmokefreeSGM is a text-based smoking cessation intervention developed to support sexual and gender minorities (SGM) with their quitting efforts. We implemented a pragmatic recruitment strategy to enroll a study sample representative for SmokefreeSGM’s feasibility trial. This study outlines the strategies employed to recruit SGM smokers in SmokefreeSGM’s feasibility trial and provides a framework for researchers interested in working with this population.

**Methods:**

This study recruited SGM smokers using ResearchMatch, social media/internet advertising, and field recruitment in the United States from January to June 2023, using convenience sampling. We evaluated enrollment rates across recruitment methods and various demographic characteristics such as age, gender identity, sexual orientation, etc. Recruitment (those who completed first part of screening divided by those who contacted us) and enrollment (those enrolled divided by those who completed first part of screening) rates were calculated.

**Results:**

The recruitment rate for this feasibility trial was 20.5% (156/761), and the enrollment rate was 50.6% (79/156). Overall, the ResearchMatch recruitment strategy resulted in the enrollment of the largest proportion, 53.2% (*n* = 42). We enrolled a greater proportion of participants who were gay and bisexual men and participants who identified as male through field recruitment. Through the social media and internet advertising modality, the highest proportion of transgender individuals were enrolled (*p* = 0.012).

**Conclusions:**

Insights gained from this experience will play pivotal role in researchers’ ability to recruit SGM participants in future randomized controlled trials and help overcome barriers to recruitment for SGM individuals in tobacco prevention and control research.

*Trial registration*: NCT05645354.

## Introduction

The prevalence of cigarette smoking among sexual and gender minority (SGM) groups (36%)—which includes but is not limited to lesbian (24%), gay (28%), bisexual (35%), transgender (32%), queer (26%), intersex, and asexual (LGBTQIA+) individuals—is significantly higher than among heterosexual and cisgender individuals (16%) [1–5]. The rates of electronic nicotine delivery system (ENDS) use have also been found to be higher in SGM adults compared to heterosexual adults [6, 7]. As a result, this population is at an increased risk for developing tobacco-related health conditions such as cancer, heart disease, stroke, lung diseases, diabetes, and chronic obstructive pulmonary disease (COPD), among others [8].

Although research on SGM youth and young adults has grown considerably over the past decade, much of this work has primarily focused on sexual health (e.g., HIV prevention) [9–11]. The lack of representation of SGM participants in tobacco-related research studies currently limits the generalizability of findings and interventions tailored to the needs of SGM participants. There are multiple determinants related to the underrepresentation of SGM in tobacco intervention research, including sexual orientation and gender identity (SOGI) concealment, stigma and discrimination, trust and SOGI disclosure, barriers to healthcare access, intersectionality, heterogeneity within SGM communities, as well as limited culturally sensitive and tailored recruitment methods [12]. Therefore, pragmatic recruitment strategies are necessary to acquire a sample that represents the population that will benefit from an intervention [13].

Engaging SGM groups through Research panels and platforms, such as Qualtrics, Dynata, ResearchMatch, CloudResearch, Prolific, and Amazon Mechanical Turk (MTurk), among others, offers a set of distinct advantages and challenges in the recruitment process for a smoking cessation intervention [14–17]. One of the primary benefits is the potential for rapid and widespread reach to a diverse range of SGM individuals, overcoming geographical constraints and facilitating a larger sample size, particularly during and after the COVID-19 pandemic [14–17]. Research panels also provide an efficient platform for identifying and recruiting participants based on specific demographic criteria, including sexual orientation and gender identity [17]. Moreover, the streamlined nature of these platforms allows for cost-effective recruitment, optimizing resources for a study of this nature [17]. However, challenges may arise in ensuring the authenticity of participant identities and experiences, as well as potential selection biases (i.e., digital divide) inherent in online platforms [14–17].

In addition, social media platforms for the recruitment of SGM participants present a range of advantages and challenges [18]. Existing trials with SGM populations have successfully employed this recruitment strategy, particularly through the use of geosocial networking applications [19–21]. On the positive side, social media offers an expansive and accessible avenue for engaging diverse SGM communities, offering an efficient means of reaching a large audience without the expenses associated with traditional advertising methods [18]. Moreover, the targeted advertising capabilities of social media platforms allow for the tailoring of recruitment messages to specific SGM subgroups, ensuring that outreach is culturally sensitive and resonant [18]. However, challenges include the potential for selection bias, as not all SGM individuals may be active on social media platforms [18]. Privacy concerns and ethical considerations also emerge as a challenge in social media recruitment [18]. Sensitive information shared on these platforms may be at risk, potentially leading to unintended disclosure of participants’ sexual orientation and/or gender identities [18]. Another challenge is the risk of fraudulent respondents and internet bots which could compromise data quality and results [22, 23].

Field recruitment offers a direct and personalized approach to reaching SGM communities, fostering a sense of trust and rapport [18, 19, 24]. Flyer posting in community-centric spaces allows for targeted outreach, creating awareness about the smoking cessation intervention within familiar and supportive environments [18, 19, 24]. Informal talks in community centers provide opportunities for open dialogue, addressing concerns, and building connections with potential participants [18, 19, 24]. Participation in festivals and fairs enables broad visibility, potentially reaching individuals who may not engage with traditional recruitment methods [18, 19, 24]. However, challenges include the potential for limited reach in geographically dispersed communities, the need for careful consideration of cultural nuances to ensure inclusivity, and the potential difficulty in locating research participants at follow-up [18, 19, 24].

To help reduce cigarette smoking and improve health outcomes among this population, our research team developed SmokefreeSGM, an SGM-tailored text-based smoking cessation intervention [25, 26]. Upon launching our feasibility trial, it became clear that targeting this niche population for enrollment in the study would pose a significant challenge. This paper delineates comprehensive recruitment methods used in an SGM-tailored smoking cessation intervention and explores potentially effective strategies for the enrollment of SGM groups. The insights gained from this experience offer valuable guidance for future recruitment endeavors with large samples and contribute to shaping the program’s real-world marketing strategy for the SGM population.

## Methods and materials

### Design

The trial for which SGM smokers were recruited was the SmokefreeSGM feasibility study. It is a randomized two-arm trial designed to evaluate the feasibility of a tailored smoking cessation intervention among SGM smokers. Participants were randomized to one of two arms: (1) SmokefreeTXT, an 8-week evidence-based program created by the National Cancer Institute (NCI) for the general population [25, 26] (2) SmokefreeSGM, an SGM-tailored text-based smoking cessation intervention. All participants received an 8- or 10-week supply of nicotine patches via mail. Participants were engaged with the intervention for 8 weeks and in data collection for 6 months. A detailed description of SmokefreeSGM has been published in the protocol and pilot-testing papers [25, 26].

### Participants

Individuals were required to self-identify as an SGM individual, be 18 years or older, currently (in the past 30 days) smoke every day and smoke five or more cigarettes per day, interested in quitting smoking in the next 15 days [27], have a cellphone number with an unlimited short messaging service (SMS) plan, have a US mailing and email addresses; and have positive cotinine saliva test results for biologically confirming current smoking status.

SGM smokers who did not understand English were excluded as the SmokefreeSGM program is only available in English at this time. Individuals who were found to have a prepaid cell phone (pay-as-you-go plan), a cellphone number that does not work, or a cellphone number that is registered to someone else were excluded. Potential participants with absolute contraindications for the nicotine patch were ineligible for the study.

### Recruitment strategies

**Fig. 1:**
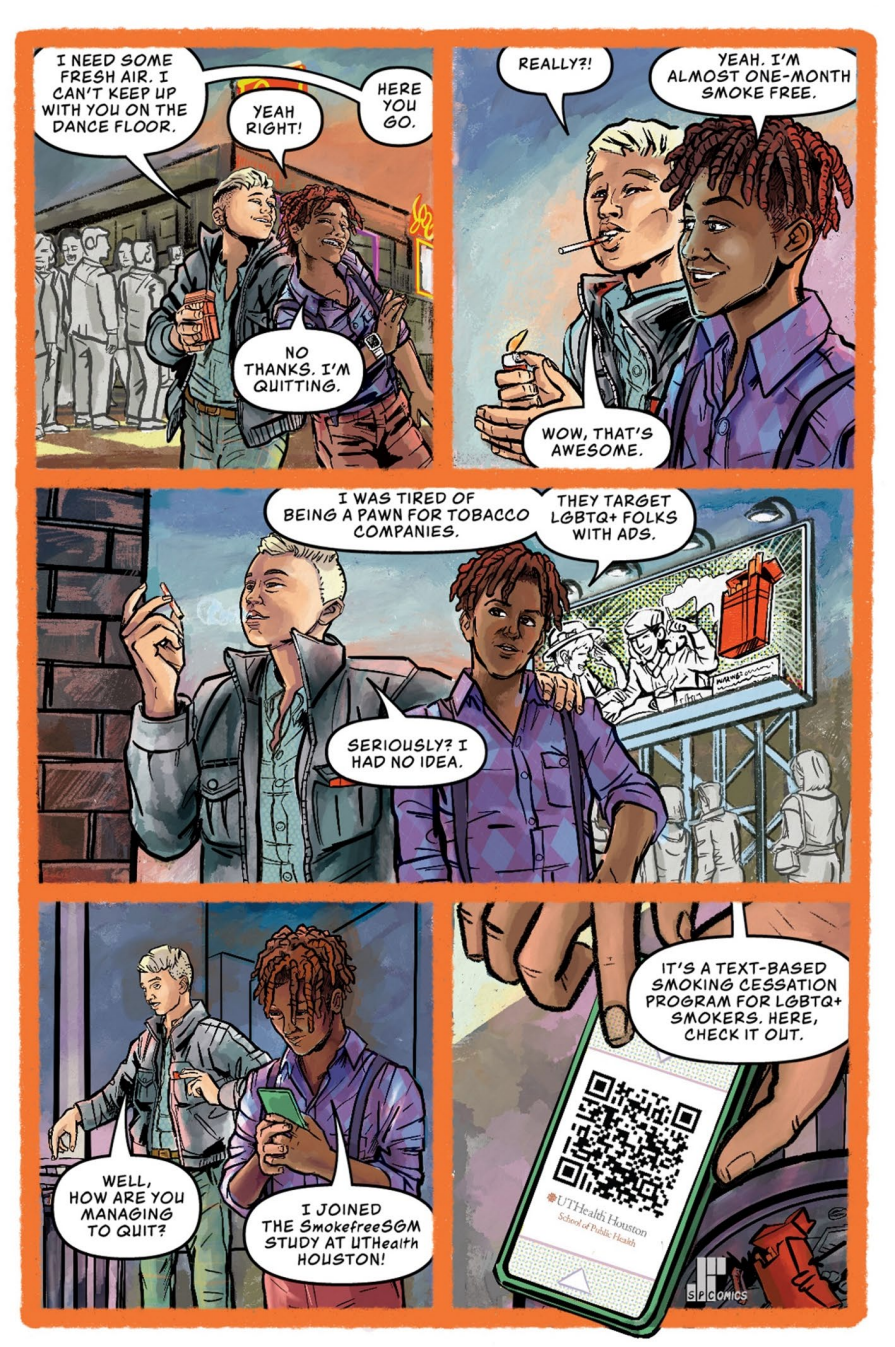
A SmokefreeSGM recruitment material (comic page) tailored to SGM smokers

Recruitment occurred from January 2023 to June 2023. This study focuses on recruitment strategies used to enroll 79 SGM smokers into the SmokefreeSGM feasibility trial. A three-pronged approach, which was comprised of ResearchMatch, social media/internet sites, and field recruitment, was used. Recruitment was earlier set for November 2022; however, the process was paused due to a glitch in the text messaging platform (one participant was recruited before the glitch). Our overarching goal was to evaluate various outreach strategies’ effectiveness rather than expedite participant enrollment. Figure 1 shows an example of a recruitment material incorporated into this study.

#### ResearchMatch

ResearchMatch, a nonprofit program funded by NIH that connects individuals interested in research studies with researchers from top medical centers across the United States (US), was used to send e-mail invitations with a brief description of the study and a link to our study form to thousands of people based on our criteria [28]. As our institution is one of ResearchMatch’s participating sites, the service was free of charge to us.

#### Social media/internet sites

We ran advertising campaigns on Facebook and Instagram, using the “boost post” feature within Facebook Ads Manager to promote our study through targeted ad sets based on age (≥ 18 years) and location (continental US), displaying concise study descriptions, a “Contact Us” form link, and our recruitment flyer across multiple placements. Additionally, we used mobile dating app for the SGM community, setting a daily budget to control impressions, and ran Google Ads campaigns with keywords like LGBTQIA+, smoking cessation, and related terms, which entered auctions for visibility until our budget was exhausted. We posted Craigslist ads in 22 metropolitan areas, adhering to posting guidelines to avoid removal, and launched email campaigns to SGM organizations nationwide, using personalized emails to encourage sharing our study through their networks, with our flyer attached and offering physical copies upon request.

#### Field recruitment

Field recruitment consisted of distributing and displaying our printed study flyer in various establishments (i.e., restaurants, coffee shops, and bars), healthcare facilities, and community organizations frequented by the SGM population across the Greater Houston metropolitan area. The flyer included information for contacting our research team by phone, email, and/or completing an electronic “Contact Us” form that a QR code could access.

### Screening and enrollment

A two-step screening procedure was implemented to enroll participants in the study and deter fraudulent or uninterested individuals from participating. Participants were contacted via phone and email. Responsive and interested participants were scheduled for Screening Part A. During Screening Part A, researchers reviewed the study’s inclusion and exclusion criteria.

Eligible participants from Screening Part A completed Screening Part B via video conference 7 days later to allow enough time to mail to their home address a saliva cotinine test (i.e., NICDetect) to verify their smoking status. A research team member monitored the saliva cotinine during the video conference. Only those individuals with positive results were eligible to participate in the study and continued to the baseline assessment.

### Measures

As part of our data collection efforts during Screening Part A, we asked potential participants about the sources through which they became aware of our study and recorded their responses. These contact modalities were categorized into three groups: (a) ResearchMatch, (b) field recruitment, and (c) social media platforms and internet sites. This allowed us to evaluate the effectiveness of our diverse recruitment efforts.

Information on the recruitment rate, screening yield rate, enrollment rate, socio-demographic characteristics, cigarette smoking, and use of electronic nicotine delivery systems (ENDS) were also collected and assessed. Recruitment rate was the proportion of individuals who contacted the research team that completed Screening Part A. Enrollment rate was the proportion of the participants who completed Screening Part A that were fully enrolled in the study i.e., those who completed Screening Part B.

Socio-demographic characteristics information collected during Screening Part A included gender identity, transgender status, sex at birth, sexual orientation, age, and participant’s state of residence. Information collected during Screening Part B included race, ethnicity, work status, level of education, marital status, whether there are children in the household, and cotinine saliva test.

### Statistical analysis

To determine the overall recruitment rate, the number of individuals who completed Screening Part A was divided by the total number of individuals who contacted our research team. For the screening yield rate for each modality, the numerator was the number of individuals who completed Screening Part A, while the denominator was the total number of flyers posted for field recruitment, the total number of email invites sent for ResearchMatch, and the total number of impressions for social media and internet sites. The overall enrollment rate was calculated by dividing the number of participants enrolled in the study (completed Screening Part B form) by the number of participants who were screened (completed Screening Part A form). A similar calculation was reproduced for each modality’s enrollment rate.

Descriptive statistics were conducted to summarize the socio-demographic characteristics of the enrolled participants. The association between the modality through which SGM smokers learned about the study and their socio-demographic characteristics was examined using Chi-squared tests (or Fisher’s exact test, when appropriate). Additionally, a multivariable binomial logistic regression model was used to assess the association between socio-demographic factors with each of the three contact modality groups, providing insights into the effectiveness of different recruitment modalities in reaching and engaging participants in the smoking cessation intervention. We ran three separate logistic regression models, with one model for each type of modality. For these regression models, the outcome variable “modality” is binary, taking on values of 1 or 0. Participants who received information about the study through a particular recruitment modality are assigned an outcome value of 1, indicating the presence of exposure to that recruitment channel. Conversely, participants who did not learn about the study through the specified modality are assigned an outcome value of 0, indicating the absence of exposure to that particular recruitment method. Due to the high collinearity between sexual orientation and gender identity, the latter was not included in the final regression models. Analysis was conducted using STATA Version 17.0 (Stata Statistical Software: College Station, TX: Stata Corp LP), and the significance level was determined using a two-sided p-value < 0.05.

## Results

### Socio-demographic characteristics

**Fig. 2:**
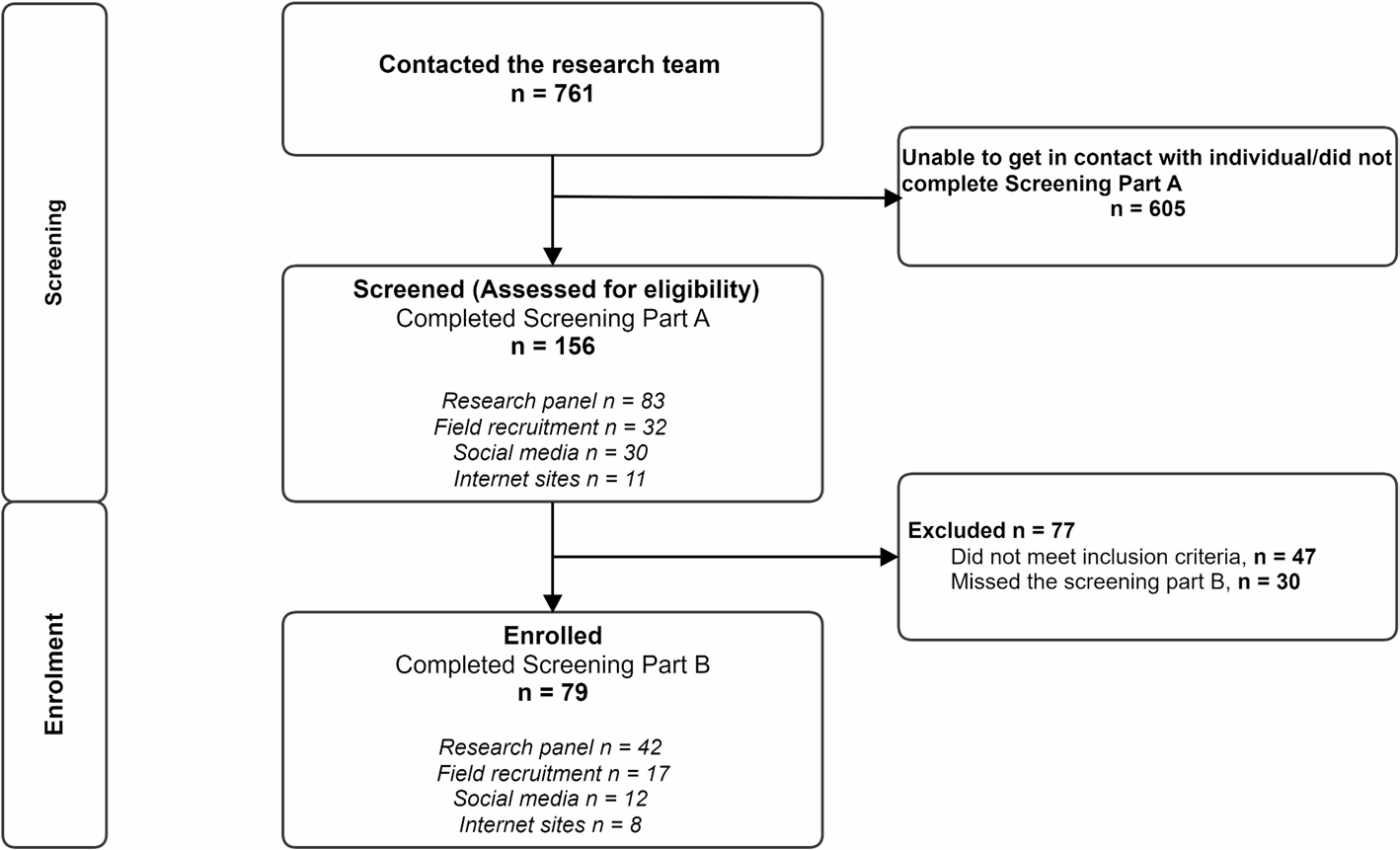
Recruitment, enrollment, and allocation flowchart

In total, 761 individuals contacted our research team, 156 completed the first screening, and 79 were enrolled in the study (see Fig. 2). We enrolled 27 gay men (34.2%), 13 lesbians (16.5%), 6 bisexual men (7.6%), 25 bisexual women (31.7%), and 8 individuals from other sexual minority groups (10.1%), such as queer, pansexual, and asexual. A majority of participants (91.1%) identified as cisgender. The gender identity of participants was 36.7% male, 49.4% female, and 13.9% transgender, non-binary, or non-gender-conforming individuals. The average age of participants was 42.3 (± 12.12) years. More than 50% of participants were non-Hispanic White (53.2%) and worked full-time/part-time (55.7%). About two-thirds of participants had completed some college or more (60.7%) and were either single, separated, divorced, or widowed (69.6%). Most participants did not have children living in their households (75.9%). (Table 1)

**Table 1:** Socio-demographic characteristics of the participants by recruitment method at baseline

Characteristics	Total *n* = 79 (100.0%)	Field recruitment *n* = 17(21.5%)	ResearchMatch *n* = 42(53.2%)	Social media/internet sites ^c^ *n* = 20 (25.3%)	*P*-value
Transgender person Yes No	7 (8.9%) 72 (91.1%)	1 (5.9%) 16 (94.1%)	1 (2.4%) 41 (97.6%)	5 (25.0%) 15 (75.0%)	**0.012** ^b^
Gender identity Male Female Transgender/non-binary/gender non-conforming	29 (36.7%) 39 (49.4%) 11 (13.9%)	9 (52.9%) 7 (41.2%) 1 (5.9%)	14 (33.3%) 24 (57.2%) 4 (9.5%)	6 (30.0%) 8 (40.0%) 6 (30.0%)	0.147b
Sexual orientation Gay/Bi-Men Lesbian/Bi-women Others	33 (41.8%) 38 (48.1%) 8 (10.1%)	8 (47.1%) 7 (41.8%) 2 (11.8%)	16 (38.1%) 23 (54.8%) 3 (7.1%)	9 (45.0%) 8 (40.0%) 3 (15.0%)	0.702^b^
Age Young (18–34 y) Middle (35–54 y) Older (> = 55 y)	28 (35.4%) 35 (44.3%) 16 (20.3%)	5 (23.5%) 10 (58.8%) 3 (17.7%)	14 (33.3%) 19 (45.2%) 9 (21.4%)	10 (50.0%) 6 (30.0%) 4 (20.0%)	0.440^b^
Race/ethnicity White Non-white	42 (53.2%) 37 (46.8%)	5 (29.4%) 12 (70.6%)	26 (61.9%) 16 (38.1%)	11 (55.0%) 9 (45.0%)	0.075^c^
Work status Yes No	44 (55.7%) 35 (44.3%)	9 (52.9%) 8 (47.1%)	25 (59.5%) 17 (40.5%)	10 (50.0%) 10 (50.0%)	0.754^c^
Education level ≤ High school diploma >High school	21 (26.6%) 58 (73.4%)	2 (11.8%) 15 (88.2%)	15 (35.7%) 27 (64.3%)	4 (20.0%) 16 (80.0%)	0.125^b^
Relationship status Single//divorced Married/with sig. other	55 (69.6%) 24 (30.4%)	14 (82.3%) 3 (17.7%)	30 (71.4%) 12 (28.6%)	11 (55.0%) 9 (45.0%)	0.184^c^
State of residence Texas Other states	37 (46.8%) 42 (53.2%)	17 (100.0%) 0 (0.0%)	4 (9.5%) 38 (90.5%)	16 (80.0%) 4 (20.0%)	**< 0.001** ^b^

^a^Includes: Facebook, Instagram, dating apps, Google Ads, Craigslist, community organizations, word of mouth

^b^ P-value from Fisher’s exact test

^c^ P-value from Chi-square test

### Recruitment and enrollment

The recruitment rate for this feasibility trial was 20.5% (156/761), and the enrollment rate was 50.6% (79/156) (Fig. 2). Resulting from the field recruitment efforts, a total of 680 flyers were distributed, 32 underwent screening (screening yield rate = 4.7%), leading to the successful enrollment of 17 participants (enrollment rate = 53.1%) through this modality. The screening process identified 83 individuals from ResearchMatch out of 1500 emails (screening yield rate = 5.5%), culminating in the enrollment of 42 participants, representing an enrollment rate of 53.9% through this modality. Our recruitment initiatives across social media and various internet platforms yielded the screening of 41 individuals from 1728 unique impressions (screening yield rate = 1.6%) and the successful enrollment of 20 participants, reflecting an enrollment rate of 48.8% **(**Table 2**).**

**Table 2:** Screening yield and enrollment rates by modality

Modality	Recruitment efforts^a^	Screening yield rate	Enrollment rate
Field recruitment ResearchMatch Social Media and Internet Sites	680^b^ 1500^c^ 1728^d^	32 (4.7%) 83 (5.5%) 41 (1.6%)	17 (53.1%) 42 (53.9%) 20 (48.8%)

^a^Different for each modality

^b^Number of flyers posted for field recruitment

^c^Number of email invites sent

^d^Total number of impressions

The ResearchMatch recruitment strategy resulted in the enrollment of the largest proportion, 53.2% (*n* = 42), of the total SGM smoker sample. Social media and internet advertisements yielded 25.3% (*n* = 20), while field recruitment yielded 21.5% (*n* = 17) of the sample. Although not statistically significant, we enrolled the highest proportion of participants who were gay and bisexual men and participants who identified as male through the field recruitment. Social media and internet advertising modality enrolled the highest proportion for transgender individuals (*p* = 0.012). Field recruitment, social media, and internet advertising were predominantly Texas participants, while ResearchMatch was predominantly participants from other states (*p* < 0.01). (Table 1)

### Predictors of enrollment by enrollment method

No statistical significance was recorded after adjusting for socio-demographic characteristics (i.e., sexual orientation, age, race/ethnicity, working status, educational level, relationship status) in the regression models for each recruitment modality.

## Discussion

Recruiting SGM populations into research is vital to the design and implementation of interventions to assist individuals addicted to tobacco products with successful quit attempts. Moreover, identifying feasible strategies that can be implemented to effectively enroll the SGM population is valuable information to researchers. This study makes important contributions to the existing body of literature on adopting innovative approaches to recruit SGM individuals who smoke into a smoking cessation intervention program aimed at helping them quit smoking. While earlier research has utilized methods such as social media to recruit SGM individuals, our study demonstrated that particular recruitment strategies can be used to recruit SGM individuals who smoke and are willing to quit. The results of this study are important as they systematically evaluate these three recruitment approaches with a side by side comparison for reaching SGM populations.

While this study did not find significant differences in the characteristics of participants recruited, it did find a difference in the number of people recruited by approach. We found that ResearchMatch was the most effective as it yielded the largest percentage of our sample. This is likely because of a research panel’s nature of reaching thousands of potential participants, more people respond and ultimately enroll in the study. Other researchers have also found this approach to be successful [15, 17]. This study found that research panels yielded double the number of participants in the sample compared to the other two recruitment strategies at no cost to the research team. This recruitment outcome indicated that researchers can utilize ResearchMatch, which may be accessible at no cost to numerous institutions in the US, to effectively recruit people who identify as sexual and gender minorities.

This study also found that social media/Internet was a successful recruitment strategy and resulted in 25.3% being recruited to this sample. This study’s recruitment approach appears to be successful because of its extensive reach and gives individuals the time to consider their participation. Others have also found this approach to be successful [18, 29]. However, just like previous studies that have used social media for recruitment among the SGM population [19, 29], this approach in our study was also at risk of negative consequences of targeted advertisement, such as cyberbullying, and the risk of fraudulent respondents. SGM populations are at risk of cyberbullying if they engage (e.g., comment section) with targeted research advertisement [30]. However, there was no history of cyberbullying in this study. In addition, the overall study design was structured to minimize the risk of fraudulent responses. For each individual, the screening process was conducted in two sequential sessions (Part A and Part B) on different days to verify that the information provided by the potential participant matched and minimize the risk of research fraud. Screening Part A and B involved a video-enabled session, during which we interacted directly with participants to confirm their eligibility for the study. While our Facebook advertisement was targeted broadly across the continental United States and our Craigslist postings were distributed across 22 U.S. cities, certain elements of our social media/internet sites recruitment strategy had a stronger presence in Texas. For instance, local organizations in Texas amplified our efforts by sharing recruitment materials on social media and posting flyers online. This regional amplification likely contributed to the higher representation of Texas participants recruited through this strategy.

The final recruitment strategies resulted in 21.5% of our sample being recruited. We believe that field recruitment approach was successful because our well-trained staff strategically place the recruitment flyers in places frequently visited by SGM individuals and also created dialogue with prospective participants and answered their questions about the study, when necessary. Our finding was consistent with the result of studies conducted among different diverse populations using a combination of antiquated recruitment strategy (such as flyers and field recruitment) and contemporary approaches (such as social media and online platform) [31–33]. Similar to these studies [31–33], field recruitment through the use of flyers resulted in the enrollment of the least number of participants. This further reinforces the significance of the use of contemporary approaches in recruiting SGM individuals who are members of a community with a relatively younger population compared to the general population.

## Limitations and strengths

As with all studies, there are limitations to the research. A key limitation of this study is that all recruitment strategies were implemented simultaneously, making it impossible to isolate and accurately measure the individual impact of each strategy. Future research should consider using distinct sources (e.g., separate URLs, email addresses, or phone numbers) to track the effectiveness of each recruitment modality. Another limitation is that, while we compare the outcomes of each recruitment method, we did not provide a detailed, side-by-side cost comparison. In general, field-based recruitment is likely more expensive due to staff salary, training, and time spent in the field, whereas social media and ResearchMatch recruitment typically involve a flat fee. Additionally, we found that there were low costs for using advertising on social media and internet sites, but at the time of the study, the institutional approval process for implementing these efforts was laborious. One other limitation to note is that certain recruitment efforts may have exhibited a delayed effect and would have gone undetected in this study’s evaluation of strategies. For example, individuals might have encountered our study advertisements but waited to contact our research team about enrollment. The time delay could mean that the study was full, and when they did contact us, we did not record it because we were no longer recruiting. Due to the small sample size in each modality, our multivariable regression models might be underpowered to detect any significant effect. Another limitation is that the findings from the field recruitment modality may not be generalizable to other cities in the US due to differing demographics, number of organizations, their receptiveness, and geopolitical factors. Finally, recruitment efforts for this study were conducted in the aftermath of the COVID-19 pandemic, during which studies of this nature encountered significant difficulties in participant recruitment. Nonetheless, recruitment efforts continued despite these substantial challenges. There is a possibility of self-selection bias, as SGM smokers who elected to participate may have been more motivated and systematically different from those who declined participation. The dichotomization of participants into ‘Texas vs. Other’ is a simplification, and the results should therefore be interpreted with caution. However, because participants outside of Texas were drawn from more than 10 different states, creating more granular categories was not feasible. Finally, we were unable to calculate recruitment rates by modality because information on how participants learned about the study was not collected when they contacted the study team. Future studies should collect this information to enable assessment of recruitment rates by modality.

Despite these limitations, the current study provides important and practical information for researchers seeking to recruit SGM populations into trials associated with smoking cessation. Enhancing practical strategies for recruitment will result in fully subscribed trials designed specifically for SGM populations.

## Conclusion

Although the recruitment of 79 SGM individuals over a 6-month period may appear suboptimal, our primary objective with this paper was to evaluate the effectiveness of various outreach strategies rather than to expedite particpant enrollment. This study provides important and practical information for researchers seeking to recruit SGM populations into trials associated with smoking cessation. Insights gained from this experience will play a pivotal role in our ability to successfully recruit participants in a future randomized controlled trial among a larger sample, inform the marketing of this program in a real-world setting, and work towards overcoming barriers to recruitment for SGM individuals in tobacco prevention and control research and public health at-large.

## Data Availability

The data supporting the findings of this study are not publicly available at this time because data analysis is still ongoing. Data will be made available upon completion of the analyses and publication of the final results.

## Declarations

### Transparency statements

The study was pre-registered at ClinicalTrials.gov (NCT05645354). The analysis plan was not formally pre-registered. De-identified data from this study are not available in a public archive. The data supporting the findings of this study are not publicly available at this time because data analysis is still ongoing. Data will be made available upon completion of the analyses and publication of the final results.. As a result, the analytic code used to conduct the analyses presented in this study is also not available in a public archive. Materials used to conduct the study are not publicly available.
